# Traveling Towards Timeliness: The Association Between Travel Time and Wait Time for Rheumatoid Arthritis Care

**DOI:** 10.3390/healthcare13192533

**Published:** 2025-10-07

**Authors:** Xiaoxiao Liu, Alka B. Patel, Judy E. Seidel, Dianne P. Mosher, John Hagens, Deborah A. Marshall

**Affiliations:** 1 Department of Community Health Sciences, Cumming School of Medicine, University of Calgary, 3280 Hospital Drive NW, Calgary, AB T2N 4Z6, Canada; alka.patel@primarycarealberta.ca (A.B.P.); judy.seidel@albertahealthservices.ca (J.E.S.); dpmosher@ucalgary.ca (D.P.M.); john.hagens@albertahealthservices.ca (J.H.); damarsha@ucalgary.ca (D.A.M.); 2 Primary Care Alberta, Calgary, AB T2W 1S7, Canada

**Keywords:** access to care, rheumatoid arthritis, wait time, travel time, primary care

## Abstract

**Objectives**: The aim was to measure wait times for rheumatologist consultation and disease-modifying antirheumatic drug (DMARD) treatment and examine their association with travel time to primary care practitioners (PCP) and rheumatologists within a centralized intake system, respectively. **Methods**: Within a centralized intake system serving 4.2 million people, we measured wait time for rheumatologist consultations and DMARD treatment for an RA incidence cohort between 1 April 2015 and 31 March 2020. Wait times were reported as the median with the interquartile range (IQR). Using multivariate logistics regression models, we examined the impact of travel times to primary/rheumatology care on wait times for rheumatologist consultation (28-day benchmark) and DMARD treatment (14-day benchmark). Travel times were defined according to quantiles and pre-defined categories. **Results**: The median wait time was 47 days (IQR: 18–114) for rheumatologist consultations (36% meeting the benchmark) and 35 days (IQR: 1–132) for DMARD treatment (43% meeting the benchmark). Patients living >120 min away had lower odds of meeting the 28-day consultation benchmark compared with those within 30 min (OR 0.64; 95% CI: 0.42–0.97). Compared with patients driving ≤30 min, lower odds of meeting the 14-day benchmark for DMARD treatment were observed for those driving over 60 min to PCPs (OR 0.62; 95% CI: 0.39–0.99) and patients driving 30–60 min to rheumatologists (OR 0.68; 95% CI: 0.55–0.85). **Conclusion**: RA management was suboptimal due to low rates of meeting RA consultation and treatment benchmarks, which was significantly associated with long travel times to both primary and RA care within a centralized triage system. This highlights the need for complementary strategies (e.g., tele-rheumatology, travel support, or alternate care providers) to ensure timely RA care in rural and remote communities.

## 1. Introduction

Timely access to rheumatology care is crucial for the optimal management of rheumatoid arthritis (RA). Prompt specialist assessment facilitates timely diagnosis and the early initiation of treatment, which has been consistently associated with improved patient outcomes and a reduced overall burden of disease [1,2,3]. However, challenges persist in ensuring timely access to rheumatology care for people with RA in Canada due to workforce shortage and the mismatch between RA demand and supply [4,5,6,7,8]. Long wait times for rheumatology consultation have been reported in Canada, with some patients waiting more than 13 months from referral to their initial visit [7,9]. Research and clinical guidelines emphasize the significance of timely access to rheumatologist consultation and disease-modified drug (DMARD) therapy [10].

In Canada, timely access to a rheumatologist depends on patients first seeing a PCP, the PCP initiating referral promptly, and the rheumatology clinics having sufficient capacity to accommodate new patients. Delays at any of the three stages may prevent patients from receiving timely and optimal care [11]. The Canadian Rheumatology Association (CRA) and Wait Time Alliance have recommended wait time benchmarks of 28 days from referral to rheumatologist consultation and 14 days from diagnosis to initiating DMARD treatment [5,12]. Reducing waits times to specialty care has been a national priority in Canada [4,13,14], yet meeting these benchmarks remains challenging. To address this, some regions, including Alberta, have implemented centralized intake systems to streamline the referral process and reduce wait times. Evidence shows that the centralized triage of rheumatology referrals is effective in improving wait times and optimizing patients’ access to care [4,5,7]. For example, the Central Referral and Triage in Rheumatology program, launched in 2006 in Calgary, serves about 2 million people across the Central, Calgary, and Southern zones of Alberta. Long-term evaluations demonstrated that this system improved referral quality and reduced wait times for moderate and urgent referrals [15]. To monitor performance, key indicators such as wait time for rheumatologist consultations and DMARD treatment have been established [16,17].

In centralized intake systems, referrals are allocated to the next available rheumatologist by urgency, regardless of patient location. While rural patients naturally face longer travel distances due to the clustering of rheumatologists in Calgary and Edmonton, the expectation is that once urgency is accounted for, wait times (measured from referral to the appointment date) should be minimally affected by geography. However, whether geographic barriers continue to affect wait times under such systems has not been well studied. Spatial access that is typically measured as travel time/distance may have an impact on both the likelihood of RA diagnosis and the likelihood of a DMARD receipt, yet findings from prior research are inconsistent. Some studies have shown that longer driving times are associated with lower odds of RA diagnosis [18,19], whereas others suggest that longer travel times are associated with a greater likelihood of DMARD treatment and better outcomes [18]. Evidence from a broader systematic review of 108 studies across health services reported that most identified a “distance decay” effect (worse outcomes with greater distances), while a minority reported opposite or null effects [20]. These conflicting findings underscore the need for more research into the complex relationship between geography, wait times, and access to care.

The objective of this study was to assess wait times to rheumatologist consultations and DMARD treatment and to examine their associations with travel times to rheumatologists and PCPs. We hypothesized that after accounting for referral urgency, wait times within a centralized intake system would be largely independent of geographic location.

## 2. Methods

We conducted a population-based cohort study on RA incident cases from 1 April 2015, to 31 March 2020 in Alberta Canada.

### 2.1. Study Area of Alberta

**Figure 1:**
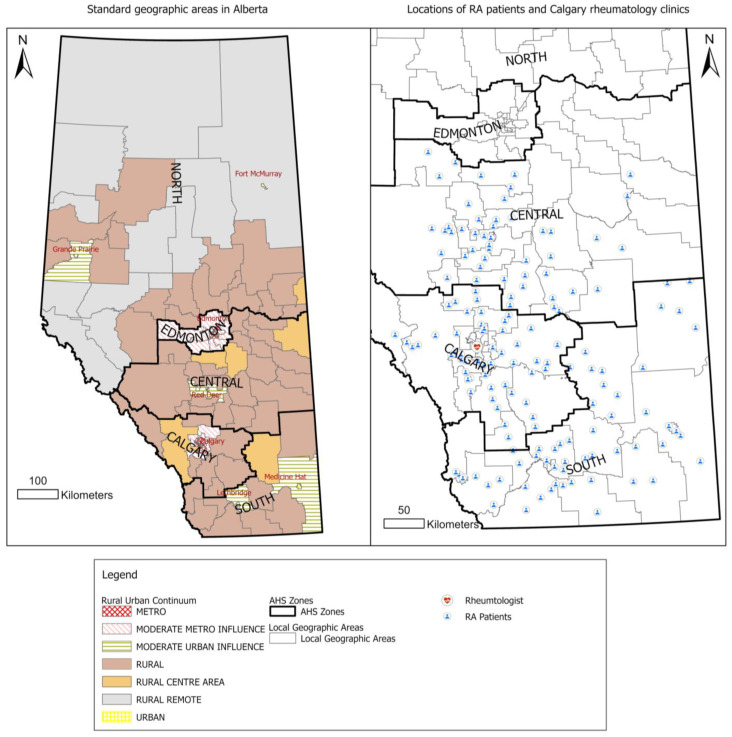
Standard geographic areas in Alberta and location of RA patients. Rheumatologists are clustered in Calgary. Patients in Calgary, Central, and Southern zones were included in the cohort.

The government (Alberta Health) and the single health authority of Alberta (Alberta Health Services—AHS) jointly created five zones in Alberta for the purpose of health service delivery, including North, Edmonton, Calgary, Central, and Southern zones (Figure 1) [21]. Considering remoteness, population density, and other factors, the province was further divided into 7 distinct rural–urban categories, known as the rural–urban continuum, encompassing Metro, Moderate Metro Influenced, Urban, Moderate Urban Influenced, Rural Centre, Rural, and Rural Remote areas [21]. In this study, we included patients from the Calgary zone, Central zone, and Southern zone only. North zone, Edmonton zone, and remote locations were excluded as they were beyond the catchment areas of the centralized triage system in Calgary.

### 2.2. RA Cohort

RA cases were identified using a validated administrative case definition previously applied in Alberta to estimate RA prevalence and incidence [22]. This case definition has a sensitivity of 83%, specificity of 99%, positive predictive value of 52%, and negative predictive value of 100% [23]. RA cases over 16 years old were identified based on one hospitalization separation or two physician claims by either PCPs or rheumatologists (at least 8 weeks apart) within a 2-year period, with ICD-9-CM codes of 714.x or ICD-10-CA codes of M05.x, M06.x [22]. The date of the second physician visit or the discharge date of hospitalization with an RA diagnosis code (whichever occurred first) was considered as the case date. Data from three administrative health databases, namely the Discharge Abstract Databases, physician claims, and the Alberta Health Care Insurance Plan (AHCIP), were linked using a unique personal identifier (a provincial health care number).

For this study, we included incident RA cases with a case date between 1 April 2015 and 31 March 2020, who remained alive and lived in Calgary, Central, or Southern zones in Alberta during the study period. This restriction ensured complete data capture for estimating wait times for rheumatology consultation and DMARD initiation. We used a run-in period from 2007/08 to 2020/21 to allow enough time to capture all prevalent cases and appropriately classify incident cases [17,24].

### 2.3. PCPs and Rheumatologists

PCPs were identified based on their specialty group designation as “GP” (General Practitioner) in the physician claims datasets. However, there is no straightforward way to differentiate between rheumatologists and internists in the claims dataset in Alberta as there is no specific identifier for rheumatologists. Previous research applied a mixed method to identify rheumatologists in Alberta [17]. We followed the same approach to create a list of rheumatologists, which includes (1) rheumatologists who self-identified as a rheumatologist and explicitly provided consent to include their personal physician identifiers for the analysis and (2) internists who had more than 20% of their claims related to RA visits between 2007/2008 and 2020/2021 [17]. This method correctly identified 93% of known rheumatologists in Alberta [17]. The centralized intake and triage program was implemented in two rheumatology clinics in Calgary providing rheumatology care to RA patients—the South Health Campus rheumatology clinic and Richmond Road rheumatology clinic (Figure 1).

### 2.4. Outcomes

The wait time for rheumatologist consultation was defined as number of days between the referral date and the rheumatologist appointment date [17]. Referrals were linked to the RA incidence cohort by unique deidentified patient IDs. Only the 1st referral was included if the patient had multiple referrals. The referral date, appointment date, and priority assessment levels—urgent, semi-urgent, and routine—were obtained from the Cerner dataset. Cerner is a scheduling tool used by Calgary rheumatology clinics to schedule patient appointments. It captures appointment scheduling data for referrals received at the Calgary Rheumatology Central Intake—South Health Campus and Richmond Road clinics. We extracted rheumatology appointments scheduled between 2012 and 2022. Wait times were categorized into two groups based on suggested benchmarks: 1 (≤28 days) and 0 (>28 days).

The wait time for DMARD treatment was defined as the number of days between the 1st RA-related physician visit and the 1st DMARD dispensing date. The Pharmaceutical Information Network (PIN) captures dates related to prescription dispensing arising from consulting with a physician. The prescription data represent the prescription filled by the patient. We obtained PIN records from 2007 to 2021 that were linked to the RA cohort using unique patient IDs. Patients with a cancer diagnosis or HIV or who were pregnant (2015/16–2019/20) were excluded. Following previous work, we included the following medications as DMARDs in our analysis: antimalarials, azathioprine, biologics, cyclophosphamide, cyclosporine, gold, leflunomide, methotrexate, minocycline, mycophenolate mofetil, penicillamine, and sulfasalazine [17]. Wait times for DMARD treatment were categorized into two groups: 1 (≤14 days) and 0 (>14 days).

### 2.5. Exposure of Interest—Travel Time to PCPs and Rheumatologists

Travel time was defined as the patients’ average driving time for PCP and rheumatologist visits, respectively, from 1 April 2019 to 31 March 2020. The driving time of each visit was calculated as the driving time between the patient’s home postal codes and provider’s clinic postal codes. Patients’ six-digit postal codes were obtained from the AHCIP population registry dataset. Providers’ six-digit postal codes were extracted from the physician claims records. Network analysis with ArcGIS Pro was applied to calculate the driving time along a digital road network [25]. The road network was modeled by AHS [26] based on the DMTI Spatial Route Logistics dataset [27]. The geocoding of both patients and providers was performed using the Alberta Health Postal Code Translator File [28].

Given the skewed distributions of wait times, patient travel time was grouped into categorical variables. Based on previous research on travel time to PCPs in Alberta, the median travel time to PCPs was 13 min (IQR: 5–28) [29]. Typically, in catchment areas of PCPs in urban and metro areas, the travel time is approximately 30 min, which may be extended to 60 min in rural remote areas. Sixty minutes has been noted as indicating a significant spatial impediment to access health care [30,31,32,33]. In this study, we employed two different categorical definitions, (1) quantiles and (2) pre-defined categories (≤30, 31–60, >60 min), to account for different utilization patterns between rural and urban patients.

Our previous study showed that the median travel time to visit rheumatologists for Albertans was 34 min (IQR:21–51), varying from 26 min (IQR: 17–36) in metro areas to 108 min (IQR: 27–141) in rural areas [29]. Considering the catchment areas with a 30 min travel time within metro areas, the 60 min travel time indicating a spatial impediment to access health care, and substantially long travel times in remote areas, we chose cut points of 30 min, 60 min, and 120 min. We applied two different category definitions, (1) quantile and (2) pre-defined categories: ≤30, 30–60, 60–120, and >120 min.

### 2.6. Other Factors

Potential confounding factors controlled for in the analysis were patient age in years, sex (male and female), referral priority assessment, provider effects, rural–urban status, and neighborhood-level median family income. The median family income—the median total income of a household in 2020—was obtained from the Canada 2021 census profile at the dissemination area (DA) level. We linked DAs with patients’ 6-digit postal codes using the Postal Code Translator File [28]. We grouped median income by quantiles (quantile 1: the lowest income).

### 2.7. Statistics

Summary statistics of the wait time for a rheumatologist consultation and wait time for DMARD treatment were reported as the median with the interquartile range (IQR), mean and standard deviation (SD), and 90% and 95% percentile. The number of RA patients meeting the wait time benchmarks was reported as numbers and the percentage of total patients within each geographic category. We applied logistics regression models to examine the association between travel times and wait times for a rheumatologist consultation and DMARD treatment. An odds ratio (OR) with a 95% confidence interval (CI) was reported. For models on the wait time for rheumatologist consultations, we ran the model using travel time to PCPs and travel time to rheumatologist consultations, respectively. For each model, we first use the travel time quantiles as exposure variables and then use the pre-defined travel time categories as exposure variables. We accounted for potential provider-level effects by including rheumatologist identifiers as fixed effects in the logistics regression model. Similarly, for models on wait time for DMARD treatment, each categorial definition of travel time to PCPs and rheumatologists were included in the model as exposure variables, respectively, to examine the relationships between travel time to providers and wait time for DMARD treatment.

### 2.8. Sensitivity Analysis

It is commonly reported that associations of driving time/distance with health outcomes tend to be sensitive to the categorial definition of travel times [20]. Sensitivity analysis was conducted by applying different categorical definitions using different travel time cut points to examine the impact of travel time definitions on the results while accounting for age, sex, median incomes, referral urgency, and provider effect.

Descriptive analyses were conducted using R 4.2.2. Network analyses were conducted using ArcGIS Pro 3.0.3.

## 3. Results

### 3.1. Summary of Wait Times for Rheumatologist Consultation 

We identified 8608 incident RA cases from 1 April 2015 to 31 March 2020, among which 65% were in Calgary, 21% in the Central zone, and 14% in the Southern zone (Table 1). Females accounted for two thirds of the cohort (5732 females vs. 2876 males). The number of cases increased from 1251 cases in 2015/16 to 2029 in 2019/20.

Among 8608 RA incident cases, 2549 (30%) cases were referred to a rheumatologist. Of those receiving RA care, 917 (36%) cases were seen by a rheumatologist within 28 days. The median wait time to see a rheumatologist was 47 days (IQR: 18–114), varying from 45 days (IQR: 14–114) in metro areas to 63 days (IQR: 28–105) in rural centres. The Calgary zone had the highest number of referrals (2119, 83%), followed by 11% in the Southern zone, and 6% in the Central zone. Calgary had the highest referral rate (38%, 2119 out 5619 cases), followed by 22% (270 out of 1280) in the Southern zone and 9% (160 out of 1781) in the Central zone (Table 2). The median wait time for urgent referrals was 24 days (IQR: 9–57), while routine referrals took four times longer (92 days, IQR: 30–190) to see a rheumatologist. The median wait times decreased from 57 days (IQR: 24–142) in 2015/16 to 35 days (IQR: 12–96) in 2018/19, though the wait time to see a rheumatologist increased again in 2019/20 (49 days, IQR: 18–117).

### 3.2. Summary of Wait Time for DMARD Treatment

As shown in Table 2, we identified 3094 (36% of RA incident cohort) RA cases who filled DMARD prescriptions, among which 43% patients (1322) received DMARD treatment within 14 days. The median wait time for DMARD treatment was 35 days (IQR: 1–132), varying from 27 days (IQR: 1–114) in rural centres to 58 days (IQR: 3–183) in rural areas. The Central zone had the shortest wait times for DMARD treatment (22 days, IQR: 1–112). The wait time for DMARD treatment presented a steady decreasing pattern, though there was a slight rise in wait times in 2019/20.

### 3.3. Association Between Travel Times and Wait Times for Rheumatologist Consultations

Using pre-defined categories for travel time to rheumatologists (≤30, 31–60, 60–120, and >120 min), we found that patients who drove over 120 min were significantly associated with a decreased odds of meeting the 28-day benchmark (OR: 0.64; 95% CI: 0.42–0.97) compared to those who drove within 30 min after accounting for age, sex, referral urgency, income, and differences among rheumatologists. Rural urban status was not included in the model due to its high correlation with travel times (Table 3). Male cases had a 29% higher odds of meeting the 28-day benchmark compared to females (OR: 1.29; 95% CI: 1.00–1.67). Urgent referrals had significantly higher odds of meeting benchmarks compared to routine referrals (urgent OR 4.84; 95% CI: 3.37–6.94; semi-urgent OR 2.05; 95% CI: 1.49–2.80). Median income had no significant association with wait times for rheumatologist consultations. Given the sensitivity of the model’s results to the different definitions of travel time, the association between travel time and wait time to see a rheumatologist was not captured using the quantile definition (Table 3).

The travel time to PCPs, modeled in either pre-defined categories (≤30, 31–60, >60 min) or quantiles, was not significantly associated with the wait time for rheumatologist consultations (Table 3). Urgent and semi-urgent referrals were found to have 4.84 and 2.03 times higher odds of meeting the 28-day benchmark than routine referrals, respectively.

### 3.4. Association Between Travel Times and Wait Times for DMARD Treatment

Predefined travel times to rheumatologists (≤30, 31–60, 60–120, >120 min) was significantly associated with wait times for DMARD treatment (Table 4). Patients who took 30–60 min to visit a rheumatologist had 32% lower odds of meeting the 14-day benchmark than the patients who lived within 30 min in terms of driving time (OR: 0.68, 95% CI: 0.55–0.85).

The predefined travel time to PCPs (≤30, 31–60, >60 min) was significantly associated with the wait time for DMARD treatment. People driving over 60 min to visit PCPs had 38% lower odds of meeting the 14-day benchmark (OR 0.62; 95% CI: 0.39–0.99) (Table 4) compared to those driving for less than 30 min.

### 3.5. Sensitivity Analysis Results

As shown in Supplementary Materials Table S1, we applied nine different definitions of travel time to rheumatologists to examine its association with wait time for rheumatologist consultations, respectively. Two definitions showed a significant association with wait times for rheumatologist consultations—the two-category definition (≤120 vs. >120 min; *p* = 0.03, OR (>120 min) = 0.64, 95% CI: 0.43–0.95) and four-category definition (≤30, 31–60, 61–120, >120 min; *p* = 0.03, OR (>120 min) = 0.64, 95% CI: 0.42–0.97). Overall, traveling over 120 min to visit rheumatologists was significantly associated with decreased odds of meeting the 28-day benchmark.

We also applied seven different definitions for travel times to PCPs. All definitions had no significant association with wait times to rheumatologist consultations.

The wait time for DMARD treatment was modelled using the nine different definitions for travel time to rheumatologists, respectively. Significant associations between travel times to rheumatologists over 30 min and the wait time for rheumatologist consultations were found in models with six different definitions, whereas the ORs ranged from 0.68 (95% CI 0.55–0.85) to 0.79 (95% CI 0.66–0.95).

The seven definitions of travel time to PCPs were included in the models of wait time for DMARD treatment. The results showed that three out of seven definitions showed significant associations with wait times for DMARD treatment, with ORs ranging from 0.60 (95% CI 0.37–0.96)–0.77 (95% CI 0.60–0.97). A longer travel time to PCPs was significantly associated with a lower probability of meeting the 14-day benchmark.

## 4. Discussion

In this paper, we assessed wait times for rheumatologist consultations and DMARD treatment from 1 April 2015 to 31 March 2020 and examined their associations with travel times to rheumatologists and PCPs within a centralized triage system. The median wait time was 47 days for a rheumatologist consultation and 35 days for DMARD treatment. The Calgary zone had the highest rate of referrals to a rheumatologist—38% RA cases were referred for RA consultations compared to 22% in the Southern zone and 9% in the Central zone. A longer travel time to see a rheumatologist was associated with decreased odds of meeting the 28-day benchmark for rheumatologist consultations and the 14-day benchmark for DMARD treatment. The driving time to PCPs had no association with wait times to see rheumatologists. Patients who took over 60 min to visit PCPs had significantly lower odds of meeting the 14-day benchmark for DMARD treatment compared to those 60 min away.

The Calgary zone had the highest percentage (38%) of RA patients being referred to a rheumatologist (2119 referred patients out of 5619 RA incident cases) (Figure 1) compared to 9% in the Central zone. This is consistent with a project report on optimizing centralized intake to improve arthritis care in Albertans [17]. The project report identified 2704 new RA cases in Alberta in the fiscal year 2015/16, among which 1021 (38%) new RAs were under the care of rheumatologists [34]. In our study, we identified 2549 out of 8608 (30%) new RA cases who had a rheumatologist appointment. The percentage of new RA cases receiving rheumatologist care among the two studies is relatively comparable though the measurement fiscal years are different. Geographical proximity and differences in the referral pattern between rural and urban physicians may contribute to this finding. Calgary patients have easy access to rheumatologists who were clustered in Calgary compared to their Southern and Central zone counterparts. Also, patients in the Central zone may prefer to go to the RA clinic in Edmonton. However, rheumatologists in Edmonton were not included in this analysis. Further research at the provincial level may help to fully understand the pattern.

We additionally compared our study with previous studies on the measurement of DMARD treatment among RA patients. Approximately 40% of new RA cases received DMARD within the fiscal year, as reported by Barber et al. 2021 [17], which is comparable to our estimate of 36% (3094 out of 8608 new RA cases from 2015/16 to 2019/20 receiving DMARD treatment). This is also consistent with the previously reported suboptimal rate of DMARD treatment in BC (37%). The low rate of DMARD treatment may be attributed reasons related to patients or the system that prevent them from filling DMARD prescriptions immediately, such as patients’ attitude towards DMARDs, their financial status, and a long waiting time to receive lab test results to ensure the safety of initiating DMARDs.

We observed a significant association between travel time to rheumatologists and wait times for rheumatologist consultations. Patients living >120 min away had lower odds of meeting the 28-day consultation benchmark compared with those who lived within 30 min (OR 0.64, 95% CI 0.42–0.97). Prior RA studies have reported inconsistent associations between spatial access and care. Some observed that longer travel times reduce the likelihood of timely RA diagnosis, while others found paradoxical results, with increased travel associated with higher odds of DMARD initiation or better outcomes [21,22]. Evidence from a broader systematic review of 108 studies indicates that most health care settings exhibit a “distance decay” effect, where patients living further away from services experience worse outcomes, although a few studies have reported opposite or null effects [20]. Given these contradictory findings, our study is important because it evaluates the impact of travel time on RA care within a centralized triage system that is designed to improve access. Rural regions have higher RA prevalence but most rheumatologists are concentrated in metro centers, resulting in substantial rural–urban disparities in travel time [29]. Similar geographic inequities have been documented in Ontario [9]. Even within the centralized triage system, patients from rural areas with long travel times may face delayed RA consultation and treatment. These findings highlight the clinical relevance of travel-related barriers and support interventions to improve access in rural and remote areas [3,35].

The results exhibited a significantly lower odds of meeting the 14-day benchmark of DMARD treatment among those who travelled over 60 min to seek care compared with those who travelled within 30 min. This finding is consistent with the previous literature. Movahedi et al. demonstrated that patients living further away from their treating clinic were significantly less likely to initiate IV biologic DMARDs [36]. Walsh et al. reported that patients living far from rheumatology sites accessed rheumatology care and DMARDs 51% and 34% less frequently than patients living close to rheumatology sites [19]. However, it is commonly reported that such relationships are sensitive to the definition of travel times. The sensitivity analysis was conducted using nine different definitions for travel time to rheumatologists and seven definitions for travel time to PCPs, which showed the robustness of association between increased travel times and decreased odds of meeting DMARD treatment benchmarks. Referral processes, waitlist management practices, and resource allocation strategies within the health care system may contribute to delays in accessing DMARD treatment, especially for those living in areas with limited access to primary and RA care. Further research is necessary to gain a comprehensive understanding of the underlying factors contributing to wait times for DMARD treatment in patients with RA. This highlights the importance of reducing geographic barriers to both primary and RA care to improve access to RA treatment by providing telehealth, travel support, and additional providers.

Our findings contribute to a broader understanding of how spatial access affects rheumatology care. Within the context of Alberta’s centralized triage system, which is designed to allocate referrals to the next available rheumatologist by urgency instead of geography, we hypothesized that travel time would have a minimal impact on wait times. However, our results demonstrate that longer travel times remained significantly associated with delays in both rheumatology consultation and DMARD initiation. Factors such as patients’ reluctance or inability to travel long distances, logistical barriers (e.g., weather, transportation availability) or differences in local health system capacity may continue to impact timely access to RA care. Clinically, these delays are highly relevant as early diagnosis and treatment are crucial for optimal RA. From a broader perspective, these findings align with concepts in spatial economics, which emphasize how geographic distance can impose costs and frictions that affect access to care. While our study is empirical in focus, it provides real-world evidence that complements theoretical work by showing how such geographic frictions persist within systems designed to streamline referrals and improve wait times. These findings indicate that while centralized intake systems improve overall access, they may not be sufficient to address geographic inequities in care. This underscores the need for complementary strategies, such as expanding outreach services, virtual care, or targeted support for rural populations, to ensure timely access to RA management.

Although the odds ratios may appear modest at first glance, their translation into absolute terms highlights clinically meaningful effects. For rheumatology consultations, with a baseline of 36% meeting the 28-day benchmark for those living within 30 min away, the OR of 0.64 translates to 26.5% meeting the benchmark among those living >120 min away. For DMARD initiation, with a 43% baseline meeting the 14-day benchmark, the OR of 0.68 corresponds to 33.9% meeting the benchmark among those living 30–60 min away. Both showed an absolute reduction of 9 percentage points in the probability of meeting the benchmark; that is, 9 fewer patients per 100 will meet the benchmark if they live further away. When scaled to provincial populations, the cumulative impact of these delays becomes substantial, suggesting a meaningful population-level impact.

This study exhibits several notable strengths. We identified RA cases using administrative health databased on a validated RA case definition, which enabled a population-based study to examine access to RA care among a large population of 4.2 million. This definition has been validated within both primary care and rheumatology care settings. A long run-in period from 2007/08 to 2020/21 was used to ensure accurate and stable estimates of RA incidence [17,24]. This algorithm is applied by PHAC in their surveillance definitions across Canada and is used in Alberta to evaluate RA burdens and estimate the performance of a centralized triage system [37]. Additionally, we used utilization data to measure travel times to care instead of assuming visits to the nearest physicians. This provides a relatively accurate measurement of travel times. Furthermore, this study explores the impact of travel time on wait times within a centralized intake and triage system. This approach offers valuable insights into the effectiveness of such systems in reducing geographic barriers regarding wait times and improving access to RA care. Finally, we conducted sensitivity analyses to assess the robustness of results under different definitions of travel times, ensuring the reliability of the identified associations between travel times and wait time measurements.

This study also contains certain limitations. First, given there is no unique identifier for rheumatologists in Alberta, rheumatologists were identified based on the frequency of RA billing codes. Though this method was not 100% accurate, it correctly identified 93% of known rheumatologists in Alberta [17]. Second, RA case definition using administrative data may lead to case misclassification and underestimating RA burden. For example, patients were not included in the cohort if they delayed seeking medical care for a prolonged time. Also, delayed referrals from primary care to rheumatologists may also lead to the exclusion of patients given the 2-year case definition window. To mitigate the risk of misclassification and disease underestimation, we used a validated RA case definition and long run-in period (2007/08–2020/21) to capture all prevalent RA cases and to avoid classifying prevalent RA cases as incident cases. Third, the study’s focus on the catchment areas of the central intake and triage system in Calgary leads to the exclusion of patients from Edmonton and the northern zones. This exclusion overlooks the unique challenges faced by patients in these areas, including those residing in vast rural and remote regions who may travel significant distances to seek RA care in Edmonton. Also, patients in the Central zone may travel to Edmonton to seek RA care. As a result, this study may underestimate the geographic disparities in wait times beyond the catchment area of the central triage system. Furthermore, we used six-digit postal codes to calculate travel times, which have may led to an underestimation of travel burden in rural areas as postal codes in rural locations cover large geographic distances compared to urban postal codes. Last, we used DMARD dispensation to represent DMARD use, which may overestimate the wait time for DMARD treatment.

## 5. Conclusions

RA management remains suboptimal within the centralized triage system, with delays linked to long travel times to both primary care and rheumatologists. This provides scientific evidence for policymakers and health service planners, underscoring the need for complementary strategies (e.g., tele-rheumatology, travel support, or alternate care providers) to ensure timely RA care in rural and remote communities.

## Figures and Tables

**Table 1:** Number of RA cases by geographic categories and fiscal year at incidence.

Geog. Categories/Fiscal Year at Incidence	Age at Incidence Year: Mean (SD)	RA Cases n (%)	Females n (%)	Males n (%)
Alberta	56 (17)	8608 (100%)	5732 (100%)	2876 (100%)
AHS zones				
Central	58 (16)	1781 (21%)	1107 (19%)	674 (23%)
Calgary	55 (17)	5619 (65%)	3768 (66%)	1851 (64%)
Southern	56 (17)	1208 (14%)	857 (15%)	351 (12%)
Rural–urban continuum				
Metro	55 (17)	4345 (51%)	2931 (51%)	1414 (49%)
Moderate metro influence	54 (17)	665 (8%)	440 (8%)	225 (8%)
Urban	56 (17)	978 (11%)	677 (12%)	301 (10%)
Moderate urban influence	54 (16)	222 (3%)	149 (3%)	73 (3%)
Rural centre	56 (17)	632 (7%)	399 (7%)	233 (8%)
Rural	59 (17)	1766 (21%)	1136 (20%)	630 (22%)
Fiscal Year at incidence				
201516	54 (17)	1251 (15%)	873 (15%)	378 (13%)
201617	56 (17)	1548 (18%)	1008 (18%)	540 (19%)
201718	55 (17)	1891 (22%)	1276 (22%)	615 (21%)
201819	56 (17)	1889 (22%)	1241 (22%)	648 (23%)
201920	57 (17)	2029 (24%)	1334 (23%)	695 (24%)

Note: SD denotes standard deviation of age at incidence. n refers to number of RA patients. % refers to the percentage of RA patients within each subgroup.

**Table 2:** Summary statistics of wait times for rheumatologist consultations and DMARD treatments.

Wait Time for Rheumatologists								
Geog. Categories/Priority	Number of Patients with Referral to Rheumatologist (%)	Number of Patients with Wait Time ≤ 28 Days (%)	Summary of Wait Time to See Rheumatologist (Days)					
			Median (IQR)	Mean (SD)	Min	P90	P95	Max
Alberta	2549 (100%)	917 (36%)	47 (18–114)	84 (97)	0	216	299	655
AHS zones								
Central	160 (6%)	56 (35%)	41 (19–86)	71 (80)	1	201	260	367
Calgary	2119 (83%)	776 (37%)	46 (17–116)	85 (99)	0	216	318	655
South	270 (11%)	85 (32%)	57 (20–122)	85 (87)	0	215	277	383
Rural–urban continuum								
Metro	1630 (64%)	612 (38%)	45 (17–114)	84 (99)	0	216	306	655
Moderate metro influence	266 (10%)	88 (33%)	50 (19–106)	86 (97)	1	218	318	460
Urban	177 (7%)	50 (28%)	60 (24–131)	89 (86)	1	230	270	368
Moderate urban influence	41 (2%)	19 (46%)	42 (8–128)	70 (79)	1	199	232	259
Rural centre	70 (3%)	18 (26%)	63 (28–105)	83 (87)	1	184	258	461
Rural	365 (14%)	130 (36%)	41 (19–103)	82 (95)	0	211	309	490
Priority level								
Routine	1085 (43%)	262 (24%)	92 (30–190)	126 (118)	0	295	381	655
Semi-Urgent	784 (31%)	275 (35%)	42 (19–81)	64 (70)	0	153	210	388
Urgent	680 (27%)	380 (56%)	24 (9–57)	41 (50)	0	98	126	446
Incidence Year								
201516	486 (19%)	144 (30%)	57 (24–142)	100 (109)	0	258	339	655
201617	555 (22%)	204 (37%)	45 (20–109)	85 (101)	0	226	339	574
201718	580 (23%)	195 (34%)	48 (20–111)	84 (96)	0	206	294	520
201819	476 (19%)	215 (45%)	35 (12–96)	69 (83)	0	184	246	471
201920	452 (18%)	159 (35%)	49 (18–117)	82 (90)	0	208	255	573
**Wait Time for DMARD Treatment**								
**Geog. Categories**	**Number of Patients on DMARD Treatment (%)**	**Number of Patients with Wait Time for DMARD Treatment Within 14 Days (%)**	**Summary of Wait Time to DMARD Treatment (Days)**					
			**Median (IQR)**	**Mean (SD)**	**Min**	**P90**	**P95**	**Max**
Alberta	3094 (100%)	1322 (43%)	35 (1–132)	125 (237)	0	354	576	2341
AHS zones								
Central	584 (19%)	276 (47%)	22 (1–112)	117 (239)	0	308	601	1701
Calgary	2074 (67%)	866 (42%)	37 (1–131)	123 (231)	0	354	555	2341
South	436 (14%)	180 (41%)	40 (1–155)	143 (263)	0	386	612	1754
Rural–urban continuum								
Metro	257 (8%)	100 (39%)	46 (1–119)	114 (196)	0	345	519	1325
Moderate metro influence	336 (11%)	146 (44%)	34 (1–135)	134 (264)	0	371	636	1754
Urban	86 (3%)	39 (45%)	28 (1–143)	130 (278)	0	268	421	1701
Moderate urban influence	193 (6%)	80 (42%)	40 (1–188)	163 (273)	0	549	711	1456
Rural centre	626 (20%)	285 (46%)	27 (1–114)	114 (226)	0	300	574	1657
Rural	479 (15%)	181 (38%)	58 (3–183)	183 (343)	0	548	932	2341
Incidence Year								
201516	479 (15%)	181 (38%)	58 (3–183)	183 (343)	0	548	932	2341
201617	576 (19%)	246 (43%)	32 (1–133)	137 (267)	0	372	696	1773
201718	686 (22%)	309 (45%)	28 (1–124)	120 (233)	0	312	547	1524
201819	677 (22%)	305 (45%)	25 (1–126)	106 (182)	0	319	520	1159
201920	676 (22%)	281 (42%)	41 (1–117)	97 (146)	0	296	435	852

Note: % refers to the percentage of RA patients within each subgroup. IQR: interquartile range. SD: standard deviation. Min: minimum of wait times. P90: 90% percentile of wait times. P95: 95% percentile of wait times. Max: maximum of wait times.

**Table 3:** Association between wait time for rheumatologist appointment and travel time to rheumatologist consultation (upper) and travel time to PCPs (lower).

Wait Time for Rheumatologist Consultation~Travel Time to Rheumatologist Consultation					
Variables	Coefficients	Std. Error	Z Value	*p* Value	OR (95% CI)
Age (Years)	0.00	0.00	1.05	0.29	1.00 [1.00, 1.01]
Sex (ref. group: female)					
Sex_Male	0.26	0.13	1.97	0.05	1.29 [1.00, 1.67]
Median Family Income (ref. group: quartile 1)					
Quantile 2	0.23	0.18	1.33	0.18	1.26 [0.89, 1.78]
Quantile 3	0.02	0.18	0.10	0.92	1.02 [0.72, 1.44]
Quantile 4	0.04	0.18	0.24	0.81	1.04 [0.73, 1.49]
Referral priority level (ref. group: routine)					
Urgent	1.58	0.18	8.57	0.00	**4.84 [3.37, 6.94]**
Semi-Urgent	0.72	0.16	4.47	0.00	**2.05 [1.49, 2.80]**
Travel time to rheumatologists (ref. group: ≤30 min)					
30–60 min	0.04	0.14	0.31	0.75	1.05 [0.79, 1.38]
60–120 min	−0.09	0.20	−0.44	0.66	0.92 [0.63, 1.35]
>120 min	−0.45	0.21	−2.11	0.03	**0.64 [0.42, 0.97]**
**Wait Time for Rheumatologist Consultation~Travel Time to PCPs**					
**Variables**	**Coefficients**	**Std. Error**	**Z Value**	***p*** **Value**	**OR (95% CI)**
Age (Years)	0.00	0.00	1.12	0.26	1.00 [1.00, 1.01]
Sex (ref. group: female)					
Sex_Male	0.25	0.13	1.92	0.06	1.28 [0.99, 1.66]
Median Family Income (ref. group: quartile 1)					
Quantile 2	0.28	0.17	1.60	0.11	1.32 [0.94, 1.86]
Quantile 3	0.10	0.17	0.55	0.58	1.10 [0.78, 1.54]
Quantile 4	0.12	0.17	0.70	0.49	1.13 [0.80, 1.59]
Referral priority level (ref. group: routine)					
Urgent	1.58	0.18	8.58	0.00	**4.84 [3.38, 6.94]**
Semi-Urgent	0.71	0.16	4.43	0.00	**2.03 [1.49, 2.78]**
Travel time to PCPs (ref. group: ≤30 min)					
31–60 min	0.27	0.26	1.06	0.29	1.32 [0.79, 2.19]
>60 min	−0.12	0.13	−0.95	0.34	0.89 [0.69, 1.14]

Note: Wait times for rheumatologist consultations is defined as the number of days from the referral date to the rheumatologist appointment date. It is categorized into two groups: ≤28 days (1) and >28 days (0). Provider fixed effects were accounted for in the model. OR: odds ratio. CI: confidence interval. PCP: primary care practitioner.

**Table 4:** Association between wait time for DMARD treatment and travel time to rheumatologist consultation (upper) and travel time to PCPs (lower).

Wait Time for DMARD Treatment~Travel Time to Rheumatologists					
Variables	Coefficients	Std. Error	Z Value	*p* Value	OR (95% CI)
Age (Years)	0.01	0.00	3.92	0.00	**1.01 [1.01, 1.02]**
Sex (ref. group: female)					
Sex_Male	0.01	0.09	0.06	0.95	1.01 [0.84, 1.21]
Median Family Income (ref. group: quartile 1)					
Quantile 2	0.02	0.12	0.15	0.88	1.02 [0.80, 1.29]
Quantile 3	0.08	0.12	0.68	0.50	1.09 [0.85, 1.39]
Quantile 4	−0.12	0.13	−0.90	0.37	0.89 [0.69, 1.15]
Travel time to rheumatologists (ref. group: ≤30 min)					
30–60 min	−0.38	0.11	−3.46	0.00	**0.68 [0.55, 0.85]**
60–120 min	−0.02	0.13	−0.18	0.86	0.98 [0.75, 1.27]
>120 min	−0.17	0.12	−1.40	0.16	0.84 [0.66, 1.07]
**Wait Time for DMARD Treatment~Travel Time to PCPs**					
**Variables**	**Coefficients**	**Std. Error**	**Z Value**	***p*** **Value**	**OR (95% CI)**
Age (Year)	0.01	0.00	3.89	0.00	**1.01 [1.01, 1.02]**
Sex (ref. group: female)					
Sex_Male	0.01	0.09	0.06	0.95	1.01 [0.84, 1.21]
Median Family Income (ref. group: quartile 1)					
Quantile 2	0.00	0.12	0.03	0.97	1.00 [0.79, 1.27]
Quantile 3	0.04	0.12	0.36	0.72	1.04 [0.82, 1.33]
Quantile 4	−0.21	0.12	−1.73	0.08	0.81 [0.64, 1.03]
Travel time to PCPs (ref. group: ≤30 min)					
31–60 min	−0.08	0.13	−0.61	0.54	0.93 [0.72, 1.19]
>60 min	−0.48	0.24	−2.00	0.05	**0.62 [0.39, 0.99]**

Note: Wait times for DMARD treatment is defined as the number of days from the 1st RA visit to the DMARD dispensing date. It is categorized into two groups: ≤14 days (1) and >14 days (0). OR: odds ratio. CI: confidence interval. PCP: primary care practitioner.

## Data Availability

The data that support the findings of this study are available from Alberta Health Services, but restrictions apply to the availability of these data, which were used under license for the current study and so are not publicly available. Data are, however, available from the authors upon reasonable request and with permission from Alberta Health Services.

## Supplementary Materials

The following supporting information can be downloaded at https://www.mdpi.com/article/10.3390/healthcare13192533/s1, Table S1: Sensitivity analysis of different travel time definitions and their associations with wait times for rheumatologist consultations and DMARD treatment.

## References

[1] Widdifield J., Paterson J.M., Bernatsky S., Tu K., Thorne J.C., Ivers N., Butt D., Jaakkimainen R.L., Gunraj N., Ahluwalia V. (2014). Access to Rheumatologists among Patients with Newly Diagnosed Rheumatoid Arthritis in a Canadian Universal Public Healthcare System. BMJ Open.

[2] Bombardier C., Hawker G., Mosher D. (2011). The Impact of Arthritis in Canada: Today and Over the Next 30 Years.

[3] Lennep D.S., Crout T., Majithia V. (2020). Rural Health Issues in Rheumatology: A Review. Curr. Opin. Rheumatol..

[4] Vogel L. (2020). How Can Canada Improve Worsening Wait Times?. Can. Med. Assoc. J..

[5] Farrer C., Abraham L., Jerome D., Hochman J., Gakhal N. (2016). Triage of Rheumatology Referrals Facilitates Wait Time Benchmarks. J. Rheumatol..

[6] Kulhawy-Wibe S.C., Widdifield J., Lee J.J.Y., Thorne J.C., Yacyshyn E.A., Batthish M., Jerome D., Shupak R., Jilkine K., Purvis J. (2022). Results From the 2020 Canadian Rheumatology Association’s Workforce and Wellness Survey. J. Rheumatol..

[7] Carpenter T., Katz S.J. (2014). Review of a Rheumatology Triage System: Simple, Accurate, and Effective. Clin. Rheumatol..

[8] Widdifield J., Paterson J.M., Bernatsky S., Tu K., Thorne J.C., Ahluwalia V., Ivers N., Butt D., Jaakkimainen R.L., Tomlinson G. (2013). The Rising Burden of Rheumatoid Arthritis Surpasses Rheumatology Supply in Ontario. Can. J. Public Health.

[9] Widdifield J., Bernatsky S., Thorne J.C., Bombardier C., Jaakkimainen R.L., Wing L., Paterson J.M., Ivers N., Butt D., Lyddiatt A. (2016). Wait Times to Rheumatology Care for Patients with Rheumatic Diseases: A Data Linkage Study of Primary Care Electronic Medical Records and Administrative Data. CMAJ Open.

[10] Emery P., Breedveld F.C., Dougados M., Kalden J.R., Schiff M.H., Smolen J.S. (2002). Early Referral Recommendation for Newly Diagnosed Rheumatoid Arthritis: Evidence Based Development of a Clinical Guide. Ann. Rheum. Dis..

[11] Nanji J.A., Choi M., Ferrari R., Lyddell C., Russell A.S. (2012). Time to Consultation and Disease-Modifying Antirheumatic Drug Treatment of Patients with Rheumatoid Arthritis--Northern Alberta Perspective. J. Rheumatol..

[12] Ontario Rheumatology Association (2017). ORA Models of Care Legacy Report.

[13] Prime Minister of Canada Cutting Wait Times, Delivering Better Health Care in Ontario. https://www.pm.gc.ca/en/news/news-releases/2024/02/09/cutting-wait-times-delivering-better-health-care.

[14] Health Care Action Plan. https://www.albertahealthservices.ca/assets/about/aop/ahs-aop-90-report.pdf.

[15] Hazlewood G.S., Barr S.G., Lopatina E., Marshall D.A., Lupton T.L., Fritzler M.J., Mosher D.P., Steber W.A., Martin L. (2016). Improving Appropriate Access to Care with Central Referral and Triage in Rheumatology. Arthritis Care Res..

[16] Barber C.E.H., Marshall D.A., Szefer E., Barnabe C., Shiff N.J., Bykerk V., Homik J., Thorne J.C., Ahluwalia V., Benseler S. (2021). A Population-Based Approach to Reporting System-Level Performance Measures for Rheumatoid Arthritis Care. Arthritis Care Res..

[17] Barber C.E.H., Lacaille D., Faris P., Mosher D., Katz S., Patel J.N., Zhang S., Yee K., Barnabe C., Hazlewood G.S. (2021). Evaluating Quality of Care for Rheumatoid Arthritis for the Population of Alberta Using System-Level Performance Measures. J. Rheumatol..

[18] Polinski J.M., Brookhart M.A., Ayanian J.Z., Katz J.N., Kim S.C., Lii J., Tonner C., Yelin E., Solomon D.H. (2014). Relationships Between Driving Distance, Rheumatoid Arthritis Diagnosis, and Disease-Modifying Antirheumatic Drug Receipt. Arthritis Care Res..

[19] Walsh J.A., Pei S., Burningham Z., Penmetsa G., Cannon G.W., Clegg D.O., Sauer B.C. (2018). Use of Disease-Modifying Antirheumatic Drugs for Inflammatory Arthritis in US Veterans: Effect of Specialty Care and Geographic Distance. J. Rheumatol..

[20] Kelly C., Hulme C., Farragher T., Clarke G. (2016). Are Differences in Travel Time or Distance to Healthcare for Adults in Global North Countries Associated with an Impact on Health Outcomes? A Systematic Review. BMJ Open.

[21] Alberta Health Services and Alberta Health (2017). Official Standard Geographic Areas.

[22] (2025). Canadian Chronic Disease Surveillance System (CCDSS)—Case Definition. https://health-infobase.canada.ca/ccdss/data-tool/.

[23] Widdifield J., Bombardier C., Bernatsky S., Paterson J.M., Green D., Young J., Ivers N., Butt D.A., Jaakkimainen R.L., Thorne J.C. (2014). An Administrative Data Validation Study of the Accuracy of Algorithms for Identifying Rheumatoid Arthritis: The Influence of the Reference Standard on Algorithm Performance. BMC Musculoskelet. Disord..

[24] Marshall D.A., Pham T., Faris P., Chen G., O’Donnell S., Barber C.E.H., LeClercq S., Katz S., Homik J., Patel J.N. (2020). Determination of Rheumatoid Arthritis Incidence and Prevalence in Alberta Using Administrative Health Data. ACR Open Rheumatol..

[25] Fischer M.M. (2006). GIS and Network Analysis.. Spatial Analysis and GeoComputation.

[26] Alberta Facilities Distance/Time Look Up Table. 2016, 1–12.

[27] DMTI Spatial. https://www.dmtispatial.com/canmap/.

[28] Alberta Health Postal Code Translator File (PCTF). 2024.

[29] Liu X., Patel A.B., Seidel J.E., Mosher D.P., Hagens J., Marshall D.A. (2025). Informing Equitable Access to Care: A Cross-Sectional Study of Travel Burden to Primary and Rheumatology Care for People with Rheumatoid Arthritis. Int. J. Equity Health.

[30] McGrail M.R., Humphreys J.S. (2014). Measuring Spatial Accessibility to Primary Health Care Services: Utilising Dynamic Catchment Sizes. Appl. Geogr..

[31] McGrail M.R. (2012). Spatial Accessibility of Primary Health Care Utilising the Two Step Floating Catchment Area Method: An Assessment of Recent Improvements. Int. J. Health Geogr..

[32] Mcgrail M.R., Humphreys J.S. (2009). The Index of Rural Access: An Innovative Integrated Approach for Measuring Primary Care Access. BMC Health Serv. Res..

[33] Bauer J., Groneberg D.A. (2016). Measuring Spatial Accessibility of Health Care Providers—Introduction of a Variable Distance Decay Function within the Floating Catchment Area (FCA) Method. PLoS ONE.

[34] PRIHS Centralized Intake Project team Final Report of the Performance Measures/Key Performance Indicators of the Rheumatoid Arthritis Care in Alberta. 2019; Volume 2.

[35] Tagimacruz T., Bischak D.P., Marshall D.A. (2021). Alternative Care Providers in Rheumatoid Arthritis Patient Care: A Queueing and Simulation Analysis. Health Syst..

[36] Movahedi M., Joshi R., Rampakakis E., Thorne C., Cesta A., Sampalis J.S., Bombardier C. (2019). Impact of Residential Area on the Management of Rheumatoid Arthritis Patients Initiating Their First Biologic DMARD: Results from the Ontario Best Practices Research Initiative (OBRI). Medicine.

[37] Widdifield J., Barber C.E.H., Bernatsky S., Eder L., Ahluwalia V., Pope J.E., Ling V., Gozdyra P., Kuriya B., Hofstetter C. (2021). Evaluation of Rheumatology Workforce Supply Changes in Ontario, Canada, from 2000 to 2030. Healthc. Policy.

