# Supplementary material for: Traveling Towards Timeliness: The Association Between Travel Time and Wait Time for Rheumatoid Arthritis Care
Source: Healthcare (Basel). 2025 Oct 7;13(19):2533. doi: 10.3390/healthcare13192533 (PMC12524471; doi:10.3390/healthcare13192533)
Supplement: Supplementary file 1 [file healthcare-13-02533-s001.zip › healthcare-3785866-supplementary.pdf]

Supplementary Materials Table S1 Sensitivity analysis of different travel time definitions and their associations with wait times to rheumatologists consultations and DMARDs treatment

Travel time definitions		Wait time to rheumatologist consultations		Wait time to DMARDs treatment	
		p value	OR (95% CI)	p value	OR (95% CI)
Travel time to rheumatologists	Travel time to rheumatologists (minutes)	0.02	1.00 (1.00-1.00)	0.30	1.00 (1.00-1.00)
	Travel time to rheumatologists (quantiles) (ref. group: quartile 1)				
	Quantile 2	0.93	0.98 (0.71-1.38)	0.14	0.83 (0.66-1.06)
	Quantile 3	0.46	0.88 (0.63-1.23)	0.11	0.82 (0.65-1.04)
	Quantile 4	0.06	0.71 (0.50-1.01)	0.46	0.91 (0.72-1.16)
	Travel time to rheumatologists (2 categories) (ref. group: <= 30 minutes)				
	> 30 minutes	0.53	0.92 (0.73-1.18)	0.01	0.79 (0.66-0.95)**
	Travel time to rheumatologists (2 categories) (ref. group: <= 120 minutes)				
	> 120 minutes	0.03	0.64 (0.43-0.95)**	0.68	0.96 (0.77-1.18)
	Travel time to rheumatologists (3 categories) (ref. group: <= 30 minutes)				
	31 - 60 minutes	0.29	1.32 (0.79-2.19)	0.00	0.68 (0.55-0.85)**
	> 60 minutes	0.34	0.89 (0.69-1.14)	0.31	0.90 (0.73-1.10)
	Travel time to rheumatologists (4 categories) (ref. group: <= 15 minutes)				
	16 - 30 minutes	0.55	1.13 (0.76-1.68)	0.91	0.98 (0.72-1.35)
	31 - 60 minutes	0.53	1.14 (0.76-1.69)	0.01	0.67 (0.50-0.91)**
	>60 minutes	0.44	0.85 (0.56-1.29)	0.43	0.89 (0.66-1.19)
	Travel time to rheumatologists (4 categories) (ref. group: <= 30 minutes)				
	31 - 60 minutes	0.75	1.05 (0.79-1.38)	0.00	0.68 (0.55-0.85)**
	61 - 120 minutes	0.66	0.92 (0.63-1.35)	0.86	0.98 (0.75-1.27)
	>120 minutes	0.03	0.64 (0.42-0.97)**	0.16	0.84 (0.66-1.07)
	Travel time to rheumatologists (5 categories) (ref. group: <= 15 minutes)				
	16 - 30 minutes	0.54	1.13 (0.76-1.69)	0.92	0.98 (0.72-1.35)
	31 - 60 minutes	0.51	1.14 (0.77-1.70)	0.01	0.68 (0.50-0.92)**
	61 - 120 minutes	0.99	1.00 (0.62-1.61)	0.84	0.97 (0.69-1.35)
	>120 minutes	0.15	0.69 (0.42-1.15)	0.26	0.83 (0.61-1.15)
	Travel time to rheumatologists (5 categories) (ref. group: <= 30 minutes)				
	31 - 60 minutes	0.75	1.05 (0.79-1.38)	0.00	0.68 (0.55-0.85)**
	61 - 120 minutes	0.66	0.92 (0.62-1.35)	0.86	0.98 (0.75-1.27)
	121 - 180 minutes	0.06	0.57 (0.32-1.03)	0.83	0.97 (0.72-1.31)
	>180 minutes	0.18	0.70 (0.41-1.18)	0.05	0.74 (0.54-0.99)**
Travel time to PCPs	Travel time to PCPs (minutes)	0.87	1.00 (1.00-1.00)	0.11	1.00 (1.00-1.00)
	Travel time to PCPs (quantiles) (ref. group: quartile 1)				
	Quantile 2	0.07	1.32 (0.98-1.78)	0.03	0.77 (0.60-0.97)**
	Quantile 3	0.02	1.44 (1.06-1.96)	0.37	0.90 (0.71-1.14)

	Quantile 4	0.25	1.20 (0.88-1.63)	0.02	0.75 (0.59-0.95)**
	Travel time to PCPs (2 categories) (ref. group: <= 30 minutes)				
	> 30 minutes	0.36	0.88 (0.66-1.16)	0.17	0.85 (0.68-1.07)
	Travel time to PCPs (2 categories) (ref. group: <= 60 minutes)				
	> 60 minutes	0.39	0.76 (0.41-1.42)	0.05	0.62 (0.39-1.00)
	Travel time to PCPs (3 categories) (ref. group: <= 15 minutes)				
	16 - 30 minutes	0.07	1.25 (0.98-1.60)	0.30	0.90 (0.74-1.10)
	> 30 minutes	0.75	0.95 (0.71-1.28)	0.10	0.82 (0.65-1.04)
	Travel time to PCPs (3 categories) (ref. group: <= 30 minutes)				
	31 - 60 minutes	0.53	0.91 (0.67-1.23)	0.54	0.93 (0.72-1.19)
	> 60 minutes	0.37	0.75 (0.40-1.40)	0.05	0.62 (0.39-0.99)**
	Travel time to PCPs (4 categories) (ref. group: <= 15 minutes)				
	16 - 30 minutes	0.07	1.25 (0.98-1.60)	0.31	0.90 (0.74-1.10)
	31 - 60 minutes	0.94	0.99 (0.72-1.36)	0.39	0.89 (0.69-1.15)
	>60 minutes	0.53	0.82 (0.44-1.53)	0.03	0.60 (0.37-0.96)**

Note:
1. Wait times to rheumatologist consultation is defined as the number of days from referal date to the rheumatologist appointment date. It is categorized into two groups: <=28 days (1) and >28 days (0). Provider fixed effect was accounted for in the model.
2. Wait times to DMARDs treatment is defined as the number of days from 1st RA visit to the DMARDs dispensing date. It is categorized into two groups: <=14 days (1) and >14 days (0).
3. '**' denotes significance level 0.05. OR: odds ratio. CI: confidence interval. PCP: primary care practitioner.
